# Flash-Infrared-Annealing-Enabled High-Temperature Sintering of Photoanodes on Flexible Polymer Foils for Ultralight Photovoltaics

**DOI:** 10.1021/acsenergylett.5c03389

**Published:** 2025-12-16

**Authors:** David Bradford, Iacopo Benesperi, Hiroaki Jinno, Naveen Bhati, Roberto Avilés-Betanzos, François Maréchal, Gerko Oskam, Chih-Jen Shih, Michael Grätzel, Sandy Sánchez, Kevin Sivula, Marina Freitag

**Affiliations:** † 5994 Newcastle University, School of Natural and Environmental Sciences, Bedson Building, Newcastle upon Tyne NE1 7RU, U.K.; ‡ University of Turin, Department of Chemistry, NIS Interdepartmental Centre and INSTM Reference Centre, Via Quarello 15/A, 10135 Torino (TO), Italy; ¶ ETH Zürich, Institute for Chemical and Bioengineering, 8093 Zürich, Switzerland; § École Polytechnique Fédérale de Lausanne, Industrial Process and Energy Systems Engineering, 1951 Sion, Switzerland; ∥ Instituto Politécnico Nacional, Departamento de Física Aplicada, Centro de Investigación y de Estudios Avanzados, Merida 97310, Yucatan, Mexico; ⊥ Center for Nanoscience and Sustainable Technologies (CNATS), Departamento de Sistemas Físicos, Químicos y Naturales, Universidad Pablo de Olavide, 41013 Sevilla, Spain; # École Polytechnique Fédérale de Lausanne, Laboratory of Photonics and Interfaces, Institute of Chemical Sciences and Engineering, School of Basic Sciences, 1015 Lausanne, Switzerland; @ École Polytechnique Fédérale de Lausanne, Laboratory for Molecular Engineering of Optoelectronic Nanomaterials, Institute of Chemistry and Chemical Engineering, 1015 Lausanne, Switzerland

## Abstract

Ultralight photovoltaics are indispensable wherever every gram counts, from self-powered Internet of Things nodes to free-hanging greenhouse covers. In dye-sensitized solar cells (DSCs) the required 450–500 °C sintering of the mesoporous TiO_2_ (m-TiO_2_) photoanode has so far limited the use of polymer substrates. Here, we replace the furnace step with flash infrared annealing. Near-IR radiation heats the 3.5 μm m-TiO_2_ layer to 550 ± 20 °C while keeping a 12.5 μm indium-tin-oxide/polyimide foil below 170 °C with a water-cooled heat-sink. We obtain complete removal of organic binders, while the substrate sheet resistance increases modestly from 60.2 to 129.8 Ω sq^–1^. Flexible DSCs reach power conversion efficiencies of 5.10% under AM 1.5G illumination, a record value for DSCs on sub-25 μm plastics. The finished devices deliver 51× the specific power of equivalent glass devices. A cradle-to-gate life cycle assessment normalized to power-per-mass reveals order-of-magnitude reductions in several categories compared with rigid hot-plate processing. Localized IR sintering thus removes the last processing barrier for truly roll-to-roll printable hybrid solar cells.

## Graphical abstract

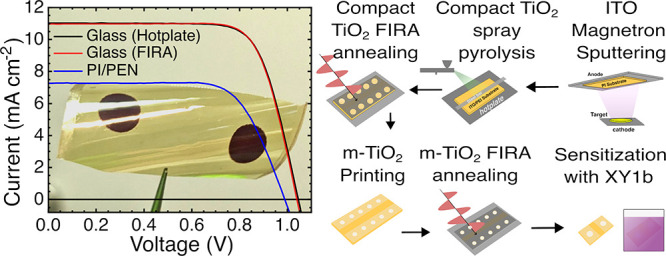

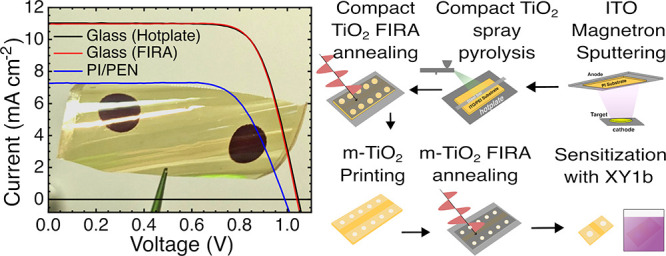

Photovoltaics are no longer judged solely by their peak efficiency; for many emerging markets the decisive metric is *power per gram*.^1^ Autonomous sensor nodes, wearable electronics, and ultralight aerial vehicles all carry stringent mass budgets, yet together they represent a projected annual demand of more than 60 GW by 2030.^2–6^ Replacing the glass sheets of a conventional dye-sensitized solar cell (DSC), which make over 80% of the device’s mass,^7^ with thin and flexible plastic substrates (FPSs) can cut transport emissions and enable integration into portable electronics and lightweight structures such as greenhouses.^8^ Furthermore, life cycle assessments (LCAs) show how FPSs have higher environmental performances compared to glass substrates (e.g., in the fossil depletion and climate change human factors categories) and lower cumulative energy demands.^7^ Achieving these environmental and weight advantages, however, requires every layer in the device stack to be deposited on polymers whose thermal stability rarely exceeds 200 °C.^9^ The critical obstacle is the sintering of the 3 μm mesoporous TiO_2_ photoanode: temperatures of 450 to 500 °C are standard on glass because they fuse particles, burn out binders, and maximize dye uptake, but they warp or melt thin FPSs in seconds. Intensive research into low-temperature pastes, chemical sintering, UV/laser treatments, and layer-transfer methods has improved the adhesion of TiO_2_ onto FPSs, yet they still trail behind the electronic quality of furnace-fired TiO_2_.^10–22^ Overcoming this mismatch between electronic performance and substrate tolerance is therefore the key materials challenge for ultralight DSCs and the wider field of roll-to-roll photovoltaics.

The use of Flash Infrared Annealing (FIRA) using Near Infrared (NIR) radiation has arisen as a sustainable, cost-efficient manufacturing method suitable for large-scale industrial production of hybrid photovoltaics.^23,24^ Induced by a set of NIR lamps, this method involves an extremely fast temperature rise and a thermal gradient between the substrate surface and the deposited films, resulting in a substantial reduction in time and energy consumption during the fabrication process. This method was demonstrated by Worsley et al., who produced highly reproducible and well-adhered sintered m-TiO_2_ films onto titanium and glass substrates in 12.5 s.^25,26^ A complete elimination of organic binders and solvents was achieved; however, the temperature reached on the substrates’ surface was very high (>545 °C), unsuitable for FPSs. More recently, Sanchez et al. demonstrated, through improved cooling systems and new short, high-intensity IR pulse protocols, the production of controlled perovskite crystallization and high-quality homogeneous perovskite films in a short amount of time.^27,28^ Furthermore, they were able to produce a thin m-TiO_2_ layer in 10 min for PSC devices and demonstrated control of the substrate temperature via rapid heat dissipation, proving FIRA’s compatibility with FPSs for DSCs.

Our work introduces a *surface-localized* FIRA process that enables, for the first time, high-temperature sintering of standard, binder-containing m-TiO_2_ films directly on tin-doped indium oxide (ITO)-coated ultrathin (12.5 μm) polyimide (PI) substrates. The FIRA setup combines high-power NIR irradiation with an actively water-cooled heat sink and a borosilicate thermal barrier, producing a steep, engineered temperature gradient: the m-TiO_2_ surface reaches 550 °C, while the area around the polymer substrate remains below 170 °C, as confirmed by real-time pyrometry and embedded thermocouples. This decoupling of the film and substrate thermal budgets, which we define as “surface-localized” heating, is not achieved in any previous large area NIR or photonic sintering approach for DSCs, as they all operate on thermally stable rigid substrates (Table S1). For what concerns flexible substrates, previous approaches (e.g., Yang et al.^17^) required cold isostatic pressing of binder-free films and multiple laser scans, highlighting the simplified and more versatile nature of FIRA. Furthermore, FIRA’s large-area illumination (35 cm^2^ per cycle) and broad process window enable scalable, high-throughput manufacturing of flexible dye-sensitized solar cells with minimal substrate degradation, in contrast to the slow, nonuniform raster scanning required for laser sintering. Achieving a power conversion efficiency up to 5.10%, the use of these FIRA-annealed PI substrates reduced the areal mass from ∼17.5 to 0.2 kg m^–2^ compared to glass and boosted the specific power from ∼5 to 255 mW g^–1^. Our LCA, when adjusted for power per unit mass density, showed relative improvements in all categories of environmental impact, with a significant relative performance increase of more than 4 orders of magnitude for the ecosystem quality and human health categories.

To produce these photoanodes, the ITO/PI substrates were thermally protected with water and gas cooling systems within the aluminum case of the FIRA equipment to prevent decomposition. As shown in Figure 1a, the TiO_2_-printed substrates were located between a 3 mm borosilicate glass spacer below and an aluminum mask above. The spacer acts as a thermal transfer retardant to the heat sink below to ensure high enough local temperatures for the TiO_2_ film to be annealed. The mask protects the non-TiO_2_-coated ITO from exposure to NIR radiation. The temperatures of the annealing programs are shown in Figures 1c and S1. The oven chamber temperatures ranged from 550 to 600 °C; in contrast, the pyrometer located at the PI sample height recorded temperatures of a maximum of 170 °C, demonstrating the efficient cooling of the heat sink and gas flow. The temperature gradient prevented the melting of the PI substrate while allowing proper annealing of the m-TiO_2_ on the unmasked surface. FTIR analysis before and after annealing, as shown in Figure 1d, revealed the elimination of peaks associated with the organic binders of the TiO_2_ paste. The sheet resistance of the ITO/PI substrate, however, increased from 60.2 to 129.8 Ω sq^–1^ (4-point probe measurement data, Figure S4) due to the thermal exposure, suggesting some degradation during annealing. By contrast, the hot-plate-annealed fluorine-doped tin oxide (FTO)-coated glass substrate maintained a comparable sheet resistance before (10.5 Ω sq^–1^) and after (11.0 Ω sq^–1^) annealing when measured with a 4-point probe instrument. The observed increase in ITO/PI sheet resistance after FIRA is not attributable to the bulk substrate temperature (which remains below 170 °C) but rather to the steep vertical temperature gradient and local overheating of the ITO layer during rapid, surface-localized annealing. This promotes microstructural changes and interfacial stress, leading to an increased resistance. Such degradation is not observed in conventional isothermal annealing at 170 °C, highlighting the unique impact of the FIRA process on the ITO/PI stability. The morphology of the sintered films was investigated using scanning electron microscopy (SEM), as shown in Figures 2 and S3. To enable direct comparison, all images were acquired at the same magnification (50k×). The hot-plate-annealed m-TiO_2_ on FTO glass (Figure 2a) exhibited a well-defined porous network of interconnected nanoparticles. Critically, the FIRA-annealed films showed a remarkably similar morphology, both on FTO/glass (Figure 2b) and on the flexible ITO/PI substrate (Figure 2c). This demonstrates that the FIRA process successfully replicated the ideal sintered structure of furnace-fired films and that the underlying substrate material did not significantly influence the resulting m-TiO_2_ morphology. The SEM cross-section displayed a photoanode with a 13.98 μm total depth, consisting of a 3.45 μm m-TiO_2_ active layer above the 10.53 μm PI film (Figures 2d and S3f). To better understand the temperature gradient in the FIRA chamber, a 2D heat transfer model was created by using the electrode layered geometry of the flexible photoanode during annealing (Figure S5). In this modeling, the PI substrate remains relatively cool, while the ITO and TiO_2_ compact layers hover over 300 °C and the m-TiO_2_ layer reaches slightly higher temperatures. These results underscore how localized heating under irradiation may support the understanding of sintering at lower bulk temperatures compared with conventional furnace-based sintering. Overall, the FIRA process was optimized to produce a 3.5 μm thick mesoporous TiO_2_ layer, a thickness considered optimal for high-efficiency DSCs employing copper-based electrolytes. This was achieved by carefully balancing NIR lamp intensity, exposure duration, and substrate cooling to ensure complete binder removal and particle necking while maintaining temperatures near the substrate below 170 °C. While thicker films are possible, they would require longer thermal exposure, necessitating further optimization to mitigate the degradation of the underlying ITO conductive layer and polymer foil.

**Figure 1:**
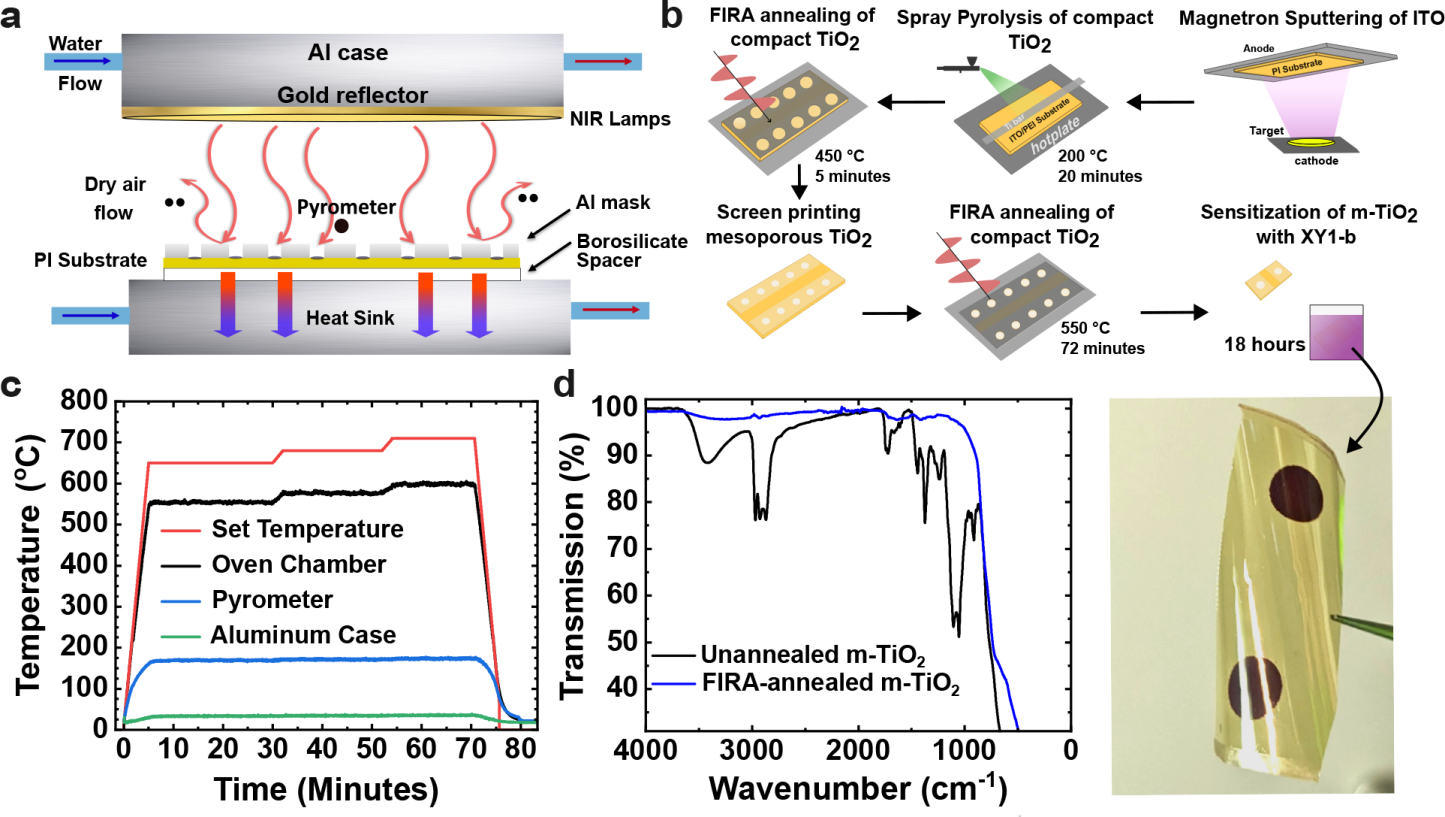
(a) Working principle schematic of the FIRA equipment. (b) Scheme of the photoanode fabrication process. (c) Temperature profile for the annealing of the mesoporous TiO_2_ film, from four temperature readings: set temperature via software, corresponding oven temperature within the chamber, pyrometer probe at sample height, and aluminum case. (d) FTIR of nonsintered and FIRA-sintered screen printed TiO_2_ film.

**Figure 2:**
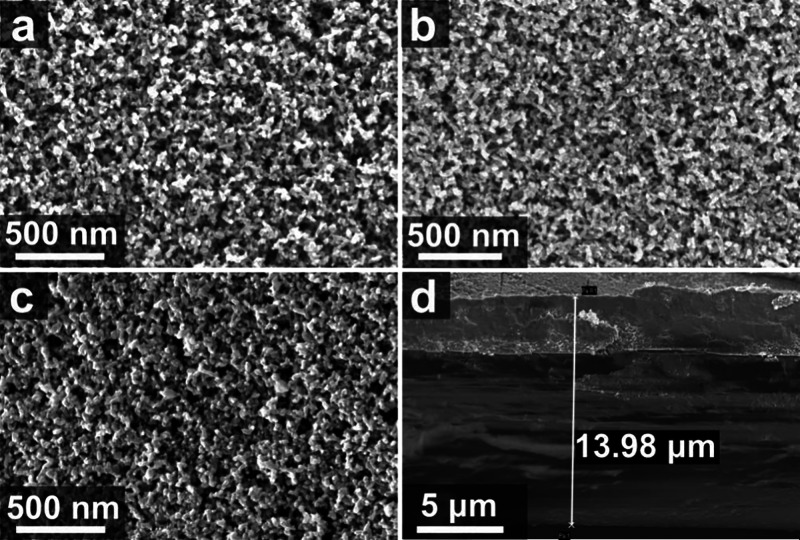
Scanning electron microscopy (SEM) images of mesoporous TiO_2_: (a) hot-plate-annealed on FTO glass (50k×), (b) FIRA-annealed on FTO glass (47k×), (c) FIRA-annealed on ITO–polyimide (50k×), and (d) cross-sectional view of an m-TiO_2_/ITO/PI photoanode (4.7k×).

After annealing, the photoanodes were immersed in a XY1b dye (Figure S2) bath for 16 h.^29^ The resulting light transmission through the film measured via UV–vis spectroscopy is presented in Figure 3a. The spectra of substrates with XY1b-sensitized m-TiO_2_ layers were similar for glass and PI. For what concerns substrates without the m-TiO_2_ layer, however, compared to glass—which showed a rapid increase in transmission starting at 314 nm—the PI substrate exhibited an increase in transmission only at higher wavelengths, starting at 416 nm. This resulted in a 32% loss of incident light available for the XY1b sensitizer to harvest in the case of the 12.5 μm PI substrate compared to glass. For comparison, a 125 μm thick PI film demonstrated an even lower transmission throughout the visible spectrum with an 83% incident light loss (compared to glass) and a transmission onset at 515 nm (Figure S6). Figure S6 also shows the red-shifted absorption of dye XY1b (λ_max_ = 531 nm)^30^ compared to Y123 (λ_max_ = 486 nm),^31^ enabling higher light harvesting through the PI substrate. This justified the adoption of the dye XY1b for the DSC devices in this work. The flexible DSC devices were assembled with a Cu­(tmby)_2_-based electrolyte (Figure S2), a 60 μm Surlyn sealant and a poly­(3,4-ethylenedioxythiophene) (PEDOT)/polyethylene naphthalate (PEN) counter electrode. In the flexible devices, the electrode spacing was reduced during the electrolyte vacuum injection step, and the 60 μm distance determined by the Surlyn sealant was not maintained. The reference (glass) DSC devices were assembled with the same electrolyte, 60 μm Surlyn sealant, and PEDOT/glass counter electrode. Glass devices labeled “FIRA” were prepared using the same annealing procedure as the PI-based m-TiO_2_, without the 3 mm glass spacer underneath.

**Figure 3:**
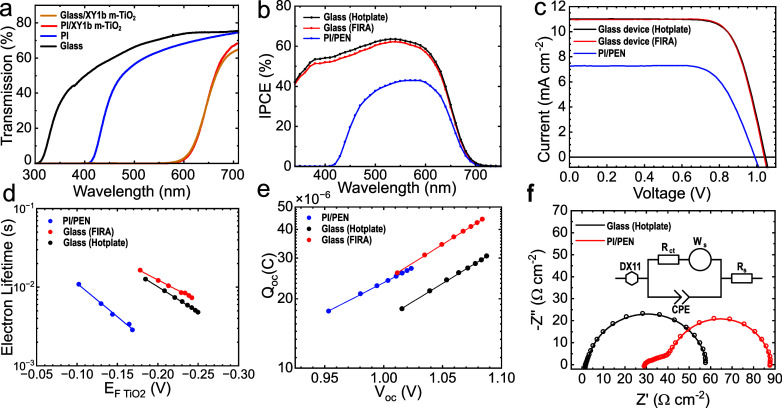
(a) UV–vis measurement of transmission through substrates without the m-TiO_2_ layer and with XY1b-sensitized m-TiO_2_ layers. (b) Incident photon-to-current efficiency spectra of the DSC devices. (c) Current–voltage characterization under AM 1.5G illumination. (d) Electron lifetime vs voltage, measured with varying light intensity. (e) Charge extraction under open circuit conditions against open circuit voltage. (f) Electrochemical impedance spectroscopy measured at *V*_OC_ under 0.15 sun-equivalent light intensity; dots: data points, line: fit to equivalent circuit model. The DX11 element represents the Bisquert transmission line element.

The photovoltaic performance of PI/PEN and glass devices was measured under AM 1.5G illumination, and the resulting current–voltage (*J*–*V*) curves are depicted in Figure 3c. *J*–*V* scan parameters open circuit voltage (*V*_OC_), short circuit current density (*J*_SC_), fill factor (FF), and power conversion efficiency (PCE) of champion devices are reported in Table 1 (complete *J*–*V* data provided in Figures S7 and S8 and Table S2). The PI/PEN device obtained a PCE of 5.10%, in comparison to the 8.82% and 8.84% of “Hot-plate” and “FIRA” glass-based devices, respectively. The *V*_OC_ of the PI/PEN device was 50 mV lower than that of the glass device at 0.99 V, indicating a lower quasi-Fermi level splitting (QFLS) of the TiO_2_. In DSCs, the electrochemical potential of electrons in TiO_2_ under open-circuit conditions is given by *E*_F,TiO_2__ = *E*_redox_ – *V*_OC_,^32,33^ where *E*_redox_ is the redox potential of the Cu­(tmby)_2_ electrolyte, as established in the literature,^34^ and is identical for all device types, as the electrolyte composition is unchanged. Therefore, any observed difference in *V*_OC_ is most reasonably attributed to a shift in the TiO_2_ quasi-Fermi level rather than a change in the electrolyte potential. This interpretation is substantiated by our experimental data, specifically electron lifetime and electrochemical impedance spectroscopy (EIS) measurements, which will be discussed later in this Letter and which indicate a reduced electron population in the TiO_2_ conduction band. As the quasi-Fermi level in TiO_2_ is a function of this electron density, these findings collectively demonstrate that the lower *V*_OC_ in the flexible device arised from a reduced QFLS in the TiO_2_. This reflected a lower light harvesting efficiency (LHE) and, together with a lower FF, higher recombination rates in devices (vide infra). The lower FF of the PI/PEN device also pointed toward higher resistances in this system (series resistance (*R*_s_) = 24 Ω cm^2^ for the PI/PEN device and 14 Ω cm^2^ for the glass device). The analysis of the *J*_SC_ reveals that the current density was significantly lower for the PI/PEN device (7.26 mA cm^–2^) compared to the glass device (11.11 mA cm^–2^). This reduction can be primarily attributed to the lower optical transmission of the PI substrate relative to glass, as evidenced by the fact that the incident photon-to-current efficiency (IPCE) of the PI/PEN device demonstrated no current generation until 410 nm because the LHE of the device was impeded by substrate absorption (Figures 3b, S9 and S10). The IPCE at each wavelength is determined by four factors, as shown in eq 1 ^35^

1 $$IPCE\left(\lambda \right)=LHE\left(\lambda \right)\times \phi_{inj}\left(\lambda \right)\times \phi_{reg}\left(\lambda \right)\times \phi_{cc}\left(\lambda \right)$$

where ϕ_inj_, ϕ_reg_, and ϕ_cc_ are electron injection, dye regeneration, and charge collection efficiencies, respectively. At the lower light intensity at which the IPCE spectrum is measured, LHE cannot be taken as the sole cause of the low intensity of the spectrum for the PI/PEN device, but there are also contributions from other factors, which are crucial for a comprehensive understanding. As better detailed later in this communication when discussing EIS results (Table S3), the PI/PEN device exhibited a higher transport resistance, a lower recombination resistance, and a lower capacitance compared to the hot-plate-annealed glass device. These parameters collectively indicate a clear impact on ϕ_cc_, thereby directly influencing the current generation of the device. This is further corroborated by the IPCE data at longer wavelengths (e.g., 600 nm), where the influence of the polyimide’s transmission becomes minimal, yet a persistent difference in current generation underscores the role of these internal device kinetics. On the other hand, a similar IPCE spectrum for the two glass devices implies that the FIRA program produced equivalent-performing m-TiO_2_ photoactive layers on rigid substrates when compared to hot-plate annealing. Despite the worse parameter values described above for the PI/PEN device compared to the FTO/glass one, the overall good charge transfer into m-TiO_2_ and charge collection of the PI photoanode was confirmed by a high shunt resistance value (21 kΩ cm^2^), which is similar to that of the glass device (12 kΩ cm^2^).

**Table 1:** Photovoltaic Parameters from *J*–*V* Characterization of Champion DSC Devices

Device	*V*_OC_ (V)	*J*_SC_ (mA cm^–2^)	FF	PCE (%)
PI/PEN	0.99	7.26	0.706	5.10
Glass “FIRA”	1.04	11.11	0.743	8.84
Glass “Hotplate”	1.05	11.16	0.734	8.82

To gain insights into charge properties in devices, transient current and voltage measurements were conducted. Electron lifetimes (τ_n_) were measured using transient photovoltage decay under small light perturbations, with decay curves fitted to a first-order exponential and τ_n_ plotted as a function of the TiO_2_ electrochemical potential. Full experimental details are provided in the Supporting Information. The electron lifetimes obtained from this measurement are portrayed in Figure 3d, which confirm the lower lifetime of the PI/PEN device with respect to glass devices. Furthermore, the charge accumulation (*Q*_OC_) against the *V*_OC_ plot, shown in Figure 3e, implied higher recombination losses and less efficient charge collection in the PI/PEN device with reference to glass devices. Interestingly, the glass “FIRA” device demonstrated the highest *Q*_OC_.^36^ Further investigation into these processes was carried out using EIS. The EIS analysis (Figure 3f) was conducted at the *V*_OC_ shown in Figure S11 (complete EIS data in Table S3). The equivalent circuit shown in Figure 3f is generally used to describe dye-sensitized solar cells,^37^ where the element DX11 corresponds to a transmission line, which includes the chemical capacitance (*C*_TiO_2__), recombination resistance (*R*_rec_), and electron transport resistance (*R*_trans_) related to the nanostructured TiO_2_ electrode. The transport of redox species in the electrolyte is captured in the Warburg element (*Z*_W_), while the charge transport resistance at the counter electrode is reflected in *R*_ct_; the CPE element describes the (leaky) capacitive properties of the counter electrode/electrolyte interface. The analysis reported a high *R*_s_ of 29.69 Ω cm^2^ (95 Ω cm^2^ extracted from the *J*–*V* curve of Figure S11) for the PI/PEN device, reflecting the degradation of the ITO. This is evidenced by the aforementioned sheet resistance of 129.8 Ω sq^–1^ of the ITO/PI post FIRA annealing. For comparison, the *R*_s_ of the hot-plate-annealed glass device was 1.70 Ω cm^2^ (38 Ω cm^2^ extracted from the *J*–*V* curve of Figure S11), with a sheet resistance of 11 Ω sq^–1^ post annealing. This highlights the dominant role of substrate conductivity in the device *R*_s_. The observed larger charge transfer resistance (*R*_ct_) of 4.59 Ω cm^2^ pointed to lower catalytic activity of the PEDOT/PEN counter electrode, in comparison to the value of 0.07 Ω cm^2^ of PEDOT/glass. These cumulative resistances resulted in a lower FF obtained for the flexible device. A recombination resistance (*R*_TiO_2__) of 41.08 and 47.50 Ω cm^2^ and a capacitance (*C*_TiO_2__) of 5.62 and 12.42 × 10^–4^ F cm^–2^ were obtained for the flexible and reference devices, respectively. This supports the observations of a lower electron lifetime and charge accumulation in the TiO_2_ conduction band. Further evidence is provided by photovoltage measurements in Figures S12 and S13, which show faster *V*_OC_ decay with regard to time and greater *V*_OC_ dependence on light intensity. The higher transport resistance (*R*_trans_) of the PI/PEN device (19.01 Ω cm^2^, glass = 0.43 Ω cm^2^) demonstrated a lower ϕ_cc_, affecting the current generation of the device. The interconnection of TiO_2_ nanoparticles and the associated trap states within the annealed mesoporous structure play a crucial role in electron transport in the semiconductor.^38^ Although SEM images show similar morphologies for the flexible film and the glass samples, these images probe only the film surface. We hypothesize that the steep thermal gradient of the FIRA process results in a vertical variation in sintering quality, where nanoparticles closer to the cooled substrate experience lower peak temperatures. This likely leads to limited interparticle connectivity and weaker necking at the buried interface, accounting for the contrasting *R*_trans_ values. The diffusion resistance (*R*_W_) for the electrolyte extracted from the Warburg component showed similar values for the glass device (8.87 Ω cm^2^) and the PI/PEN device (6.61 Ω cm^2^). These values were obtained under open circuit conditions, and since the *V*_OC_ (and thus the electron density in TiO_2_) differs between devices, direct comparison is not possible. Nonetheless, *R*_W_ remains relatively small in both cases, confirming that diffusion resistance is not a dominant factor in determining device performance. A combination of the transmission line model and Warburg element was used for the equivalent circuit to accurately extract *R*_rec_ and *R*_W_ given the frequency overlap between the photoanode and electrolyte contributions to the EIS spectrum characteristic of copper redox mediators.

To investigate the sustainability of these flexible devices, a life cycle assessment (LCA) was conducted to compare the environmental impacts of hot-plate and FIRA annealing methods. Here, a cradle-to-gate analysis was carried out with a functional unit of 1 cm^2^ for the final substrate. Moreover, only the steps that are different between the two alternatives are considered for the analysis, which highlighted only the relative differences between the two alternatives for the differing steps instead of the absolute difference for complete devices. The system boundaries for the analysis are shown in the Supporting Information (Figure S14), along with the absolute values for the environmental impacts in different scenarios (Tables S4 and S5) and further methodology details (Tables S6 and S7). Based on the analysis shown in Figure 4a, FIRA-based rigid substrates had slightly higher environmental impacts (2.23 times) compared to hot-plate-treated substrates for the TiO_2_ deposition step. However, it needs to be highlighted that flexible substrates cannot be processed with a hot-plate, as discussed above. In the second stage, FIRA-based flexible substrates were compared with FIRA-based rigid substrates, as shown in Figure 4b,c. On a geometrical area basis, flexible substrates had significantly lower environmental impacts for the ecosystem quality (EQ) and human health (HH) indicators and slightly higher impacts for the natural resources (NR) and global warming potential (GWP) indicators. Further, the impact was significantly higher (6.6 times) for the cumulative energy demand (CED) category for flexible substrates. However, when taking into account the power to mass density ratio of the substrates, the impacts for flexible substrates became significantly lower compared to those for rigid substrates. This emphasizes the potential advantage of using lightweight flexible substrates for applications that prioritize achieving higher power densities.

**Figure 4:**
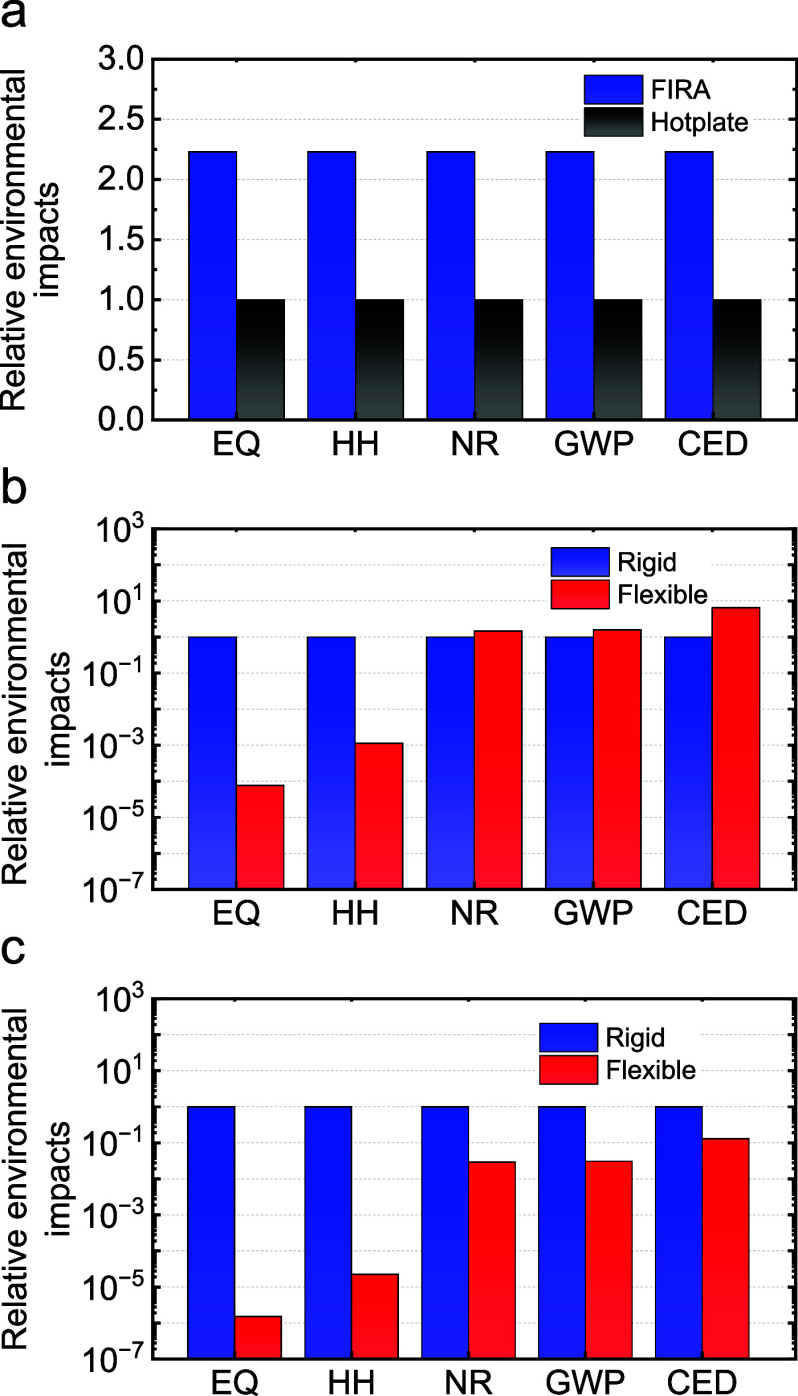
Relative environmental impacts for different processes of (a) FIRA- and hot-plate-processed rigid substrates, (b) FIRA-processed rigid and flexible substrates, and (c) FIRA-processed rigid and flexible substrates adjusted for power per unit mass density.

In conclusion, this study proved that FIRA is a viable high-temperature treatment for the fabrication of ultrathin, polymer-based DSC photoanodes. Over the course of 76 min it was possible to fully anneal and sinter a 3.5 μm thick m-TiO_2_ layer in an oven at over 550 °C, as confirmed by FTIR and SEM, while keeping the 12.5 μm PI substrate temperature below 170 °C to prevent degradation. The conductive ITO layer also experienced minimal damage, with its sheet resistance increasing from 60.2 to only 129.8 Ω sq^–1^ after the treatment. Flexible DSCs assembled with XY1b dye, a Cu­(tmby)_2_ redox shuttle, and a PEDOT/ITO/PEN counter electrode achieved up to 5.10% PCEs under AM 1.5G illumination (1000 W m^–2^) and 5.38% at 120 W m^–2^. The weight of the final device was only 200 g m^–2^, yielding a specific power of 255 mW g^–1^—51 times higher than the glass references with a photoanode 250 times thinner. Life cycle analysis normalized to W g^–1^ showed order-of-magnitude increases in performance in all categories compared to hot-plate processing. Remaining bottlenecks are optical losses from the polyimide substrate (32% photon loss in the visible range) and a 2-fold rise in ITO sheet resistance caused by damage during IR annealing. Implementing colorless substrates with lower sheet resistances and shortening the IR annealing duration are projected to lift 1-sun efficiencies beyond 8%. While the current commercial availability of colorless, high-temperature TCO-coated polymers is limited, the compatibility of FIRA with such materials represents a clear pathway for future optimization. By localizing the thermal budget to the semiconductor while preserving the polymer conductor, FIRA removes the last furnace-temperature barrier to roll-to-roll manufacture of ultralight, high-specific-power DSC foils.

## Supplementary Material



## Notes

Experimental procedures and characterization data supporting the findings of this study, including detailed photovoltaic performance metrics, SEM images, thermal modeling, spectroscopic measurements (IPCE and UV–vis), electrochemical impedance spectroscopy data and square-wave modulation measurements (PDF)
