# Supplementary material for: Flash-Infrared-Annealing-Enabled High-Temperature Sintering of Photoanodes on Flexible Polymer Foils for Ultralight Photovoltaics
Source: ACS Energy Lett. 2025 Dec 16;11(1):699–706. doi: 10.1021/acsenergylett.5c03389 (PMC12797324; doi:10.1021/acsenergylett.5c03389)
Supplement: Supplementary file 1 [file nz5c03389_si_001.pdf]

Supporting Information

Flash Infrared Annealing-Enabled High-Temperature Sintering of Photoanodes on Flexible Polymer Foils for Ultralight Photovoltaics

David Bradford[1,+], Iacopo Benesperi[2,1,+], Hiroaki Jinno[3], Naveen Bhati[4], Roberto Avilés-Betanzos[5,1], François Maréchal[4], Gerko Oskam[5,6], Chih-Jen Shih[3], Michael Grätzel[7], Sandy Sánchez[8], Kevin Sivula[8*], and Marina Freitag[1*]

[1]Newcastle University, School of Natural and Environmental Sciences, Bedson Building, Newcastle upon Tyne, NE1 7RU, UK
[2]University of Turin, Department of Chemistry, NIS Interdepartmental Centre and INSTM Reference Centre, Via Quarello 15/A, 10135 Torino (TO), Italy
[3]ETH Zürich, Institute for Chemical and Bioengineering, 8093 Zürich, Switzerland
[4]École Polytechnique Fédérale de Lausanne, Industrial Process and Energy Systems Engineering, 1951 Sion, Switzerland
[5]Instituto Politécnico Nacional, Departamento de Física Aplicada, Centro de Investigación y de Estudios Avanzados, Merida 97310, Yucatan, Mexico
[6]Center for Nanoscience and Sustainable Technologies (CNATS), Departamento de Sistemas Físicos, Químicos y Naturales, Universidad Pablo de Olavide, 41013, Sevilla, Spain
[7]École Polytechnique Fédérale de Lausanne, Laboratory of Photonics and Interfaces, Institute of Chemical Sciences and Engineering, School of Basic Sciences, 1015 Lausanne, Switzerland
[8]École Polytechnique Fédérale de Lausanne, Laboratory for Molecular Engineering of Optoelectronic Nanomaterials, Institute of Chemistry and Chemical Engineering, 1015 Lausanne, Switzerland
[+]These authors contributed equally
[*]kevin.sivula@epfl.ch
[*]marina.freitag@newcastle.ac.uk

Contents

1 Advances in Infrared Annealing of Mesoporous TiO_2 for Photovoltaic Applications 3

2 Device Fabrication 3
2.1 Flexible Photoanode 3
2.2 Glass Photoanode 4
2.3 Counter Electrode 5
2.4 Electrolyte 5
2.5 Cell Assembly 5

3 Material Analysis 6
3.1 Scanning Electron Microscopy 6
3.2 Four-Terminal Sensing 6
3.3 Thermal Modeling 7
3.4 UV-Vis Spectroscopy 8

4 Photovoltaic Characterization 9
4.1 Current Density-Voltage Data 9
4.2 IPCE and UV-Vis Transmission 11
4.3 Electrochemical Impedance Spectroscopy 12
4.4 Square-Wave Modulations Measurements under Illumination 13

5 Life Cycle Assessment 14

6 Supplemental Experimental Procedures 15
6.1 FIRA annealing 15
6.2 UV-Vis Spectroscopy 15
6.3 4-point probe 15
6.4 Current density–voltage and IPCE characterization 15
6.5 Transient Current and Voltage Measurements (“Toolbox”) 16
6.6 Electrochemical Impedance Spectroscopy 16
6.7 Scanning Electron Microscopy 16
6.8 Thermal Modeling 16
6.9 Fourier Transform Infrared (FTIR) Spectroscopy 16
6.10 Life Cycle Assessment 17

1 Advances in Infrared Annealing of Mesoporous TiO_2 for Photovoltaic Applications

Table S1: Sample details of IR-annealed m-TiO_2 layers for DSC applications present in the literature, and previous use of our FIRA equipment with perovskite solar cells.

Substrate	TiO_2 thickness	TiO_2 form	IR source Exposure time	Peak T (°C) Thermal notes	Dye, elec. Efficiency (%)	Ref.
FTO/glass	15 µm	paste	Far-IR lamp 5+10 min	250+500 two steps	D719, I_2 4.37	S1
Ti metal	unknown	paste	Near-IR lamp 12.5 s	580 to 700 back of Ti sheet	N719, I_2 2.25	S2
Ti metal	6.5 µm	paste	Near-IR lamp 12.5 s	545 back of Ti sheet	N719, I_2 2.9	S3
FTO/glass	9 µm	paste	Near-IR lamp 12.5 s	680	N719, I_2 5.2	S4
ITO/PEN	13 µm	compressed powder	1064 nm laser 30 mm s^{-1}	unknown localized heating	N719, I_2 5.7	S5
ITO/PI	3.5 µm	paste	Near-IR lamp 76 min	170 above sample	XY1b, $Cu(tmby)_2$ 5.1	this work
FTO/glass	3.5 µm	paste	Near-IR lamp 76 min	170 above sample	XY1b, $Cu(tmby)_2$ 8.8	this work
FTO/glass	200 nm	diluted paste	Near-IR lamp 10 × 15 s	600 chamber bottom	perovskite, spiro 20.1	S6

2 Device Fabrication

2.1 Flexible Photoanode

The polyimide (PI) 12.5 µm films were obtained from UBE Corporation (UPILEX 12.5SN) and coated with amorphous ITO at FIRST Lab in ETH Zurich. The ITO/PI films were cut to 3×10 centimeter substrates using a blade and heated with a hotplate to 200 °C. Steel sheets of 0.8×12.0 cm were placed over the center of the substrate, holding the lightweight substrates in place and covering a fraction of the ITO/PI. A solution of 0.1 M titanium bis(isopropoxide) bis(acetylacetonate) in isopropanol (Sigma, 600 µL per 30 cm^2 of substrate) was then deposited via spray pyrosis to form a compact TiO_2 layer. After cooling down to room temperature, the substrates were secured to 3 mm thick borosilicate glass with Kapton tape at the top and bottom edges of the substrate. The substrates were transferred to the FIRA equipment inside a dry box, and placed atop the aluminum heat sink. A 3 mm aluminum mask with 0.8 cm diameter circles (2×7) was placed over the sample. The FIRA program was set with a ramp to 550 °C over 120 seconds and held at temperature for 5 minutes as demonstrated in figure SS1. Once removed from the FIRA equipment, a mesoporous TiO_2 (m-TiO_2) layer was deposited via screen printing using a 61-63 W mesh screen (Seritec Services SA, Switzerland) and 30 NR-D titania paste (Greatcell Solar Materials, Australia). During screen printing, a supporting glass of 2 mm thickness was used, with Kapton tape, to hold substrates in place. After

printing, the 0.282 cm^2 m-TiO_2 films were rested for 5 minutes before drying at 150 °C for 10 minutes on a hotplate. Partially-dried substrates were then transfer back to the FIRA equipment to anneal the m-TiO_2. Once again, the substrates were secured on to the 3 mm borosilicate glass. This time, a 3 mm thick aluminum mask with 0.6 cm diameter circles (2×7) was placed on top of the sample and aligned to expose only the m-TiO_2 films. The set temperature for the FIRA ramped to 650 °C over 5 minutes, maintaining this temperature for 25 minutes. Then, step-wise increases to 680 and 710 °C followed, with 2 minutes ramp times, and each temperature maintained for 20 minutes. The total annealing time was 76 minutes. The annealed m-TiO_2 films were then cut to 2×1 circles pieces (see Figure 1b in the main text) and placed in a dye bath for 18 hours. The dye bath consisted of 0.1 mM XY1b dye (Dyenamo, Sweden, structure shown in Figure S2) and 2.5 mM chenodeoxycholic acid (Sigma, Switzerland) in chloroform:ethanol 3:7. Upon removal of the substrates from the dye bath, they were washed and submerged in acetonitrile.

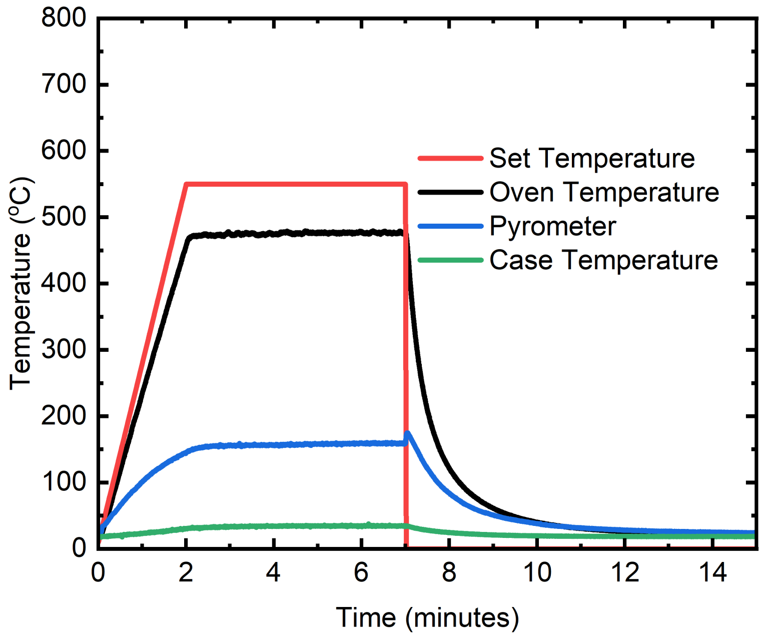

Figure S1: FIRA temperature profile of software, oven, pyrometer (above sample) and aluminum case over time of compact TiO_2 layer annealing.

2.2 Glass Photoanode

The 4 mm FTO glass (Nippon Sheet Glass Co., Ltd., 10 Ω sq^{-1} sheet resistance) substrates were washed 3 times for 30 minutes each time, immersed sequentially in 2% hellmanex detergent, deionized water, and ethanol in an ultrasonic bath. They were further cleaned in a UV-ozone chamber for 15 min before compact TIO_2 layer deposition. The glass substrates labeled "FIRA" followed the same spray pyrosis procedure as the flexible substrates, with the exception that the 3 mm borosilicate spacer was removed during FIRA annealing. The compact layer were annealed for the glass hotplate samples at 450 °C over 30 minutes. The m-TiO_2 films were deposited using the same procedure explained above; their annealing was the same as that of the flexible substrates for the "FIRA" glass, while substrates prepared via hotplate underwent a step-wise sintering program to up to 450 °C for a total of 1 hour on a programmable hotplate. Upon completion of annealing, these substrates were also cut in 2×1 pieces and the annealed m-TiO_2 films were placed in the same dye bath conditions as

those of the flexible substrates for 18 hours. Upon removal of the substrates from the dye bath, they were washed and submerged in acetonitrile.

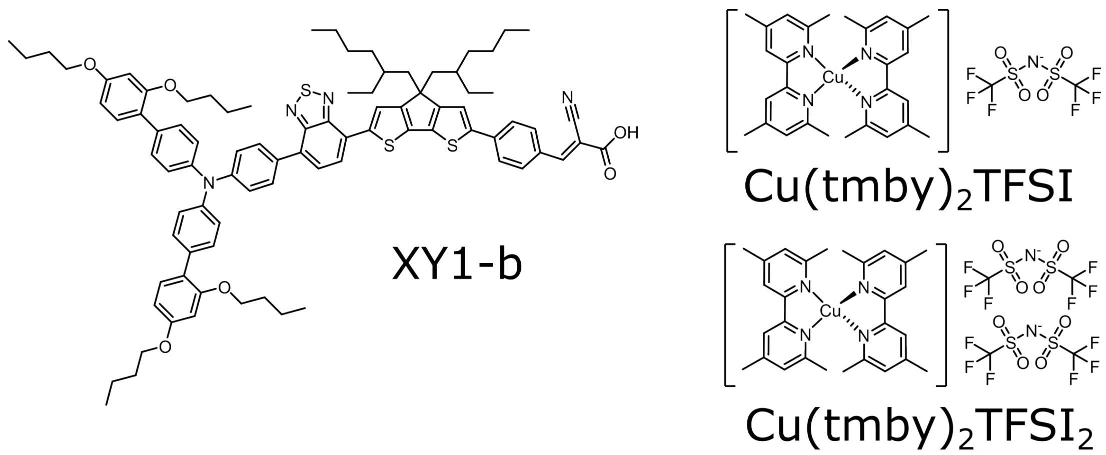

Figure S2: Molecular structures of XY1b dye, and of $[Cu(tmby)_2](TSFI)$ and $[Cu(tmby)_2](TSFI)_2$ redox mediators.

2.3 Counter Electrode

Counter electrodes for flexible cells were fabricated from 125 μm thick polyethylene naphthalate films coated with ITO, with 15 Ω sq^{-1} sheet resistance (Sigma, Switzerland). Counter electrodes for glass devices were fabricated from 3 mm FTO glass (Nippon Sheet Glass, 10 Ω sq^{-1} sheet resistance) with pre-drilled injection holes. In both cases, a film of poly(3,4-ethylenedioxythiophene) (PEDOT) was deposited electrochemically following the procedure reported in ref. S S7 using a Biologic potentiostat (Biologic, France).

2.4 Electrolyte

The electrolyte composition was constant for all devices in this study. It consisted of 0.2 M $[Cu(tmby)_2](TSFI)$, 0.09 M $[Cu(tmby)_2](TSFI)_2$ (Dyenamo, Sweden), 0.1 M NaTSFI and 0.6 M NMBI (Sigma, Switzerland) in dry acetonitrile (tmby = 4,4',6,6'-tetramethylbipyridine; TFSI = bis(trifluoromethanesulfonyl)imidate; NMBI = *N*-methylbenzimidazole. Structures of Cu complexes shown in Figure S2). The electrolyte was prepared on the same day of cell assembly.

2.5 Cell Assembly

The PI photoanodes were taped to a supporting 3 mm glass and cut to contain one active area. The PEDOT/PEN counter electrodes were also cut to size and an injection hole was punctured using a needle. 60 μm Suryln (Solaronix SA, Switzerland) frames were cut to a 2 mm width with electrolyte area of 1 cm^2. The three components were stacked on top of the support glass and backed with another 3 mm glass. Sealing was performed using a 120 °C heated press, with 2 bar pressure from compressed gas, for 60 seconds. The resulting cells were then injected with a 10 μm pipette and sealed with UV curing glue (ThreeBond 3035B). This second sealing with UV glue around the edge of the Suryln was performed to strengthen the adhesion of the two electrodes. Silver contacts were painted on the two electrodes using silver conductive paint (RS components, UK). The procedure for glass-based devices was identical, but excluded the use of a supporting glass and tape during sealing, and the injection holes were pre-drilled by the supplier.

3 Material Analysis

3.1 Scanning Electron Microscopy

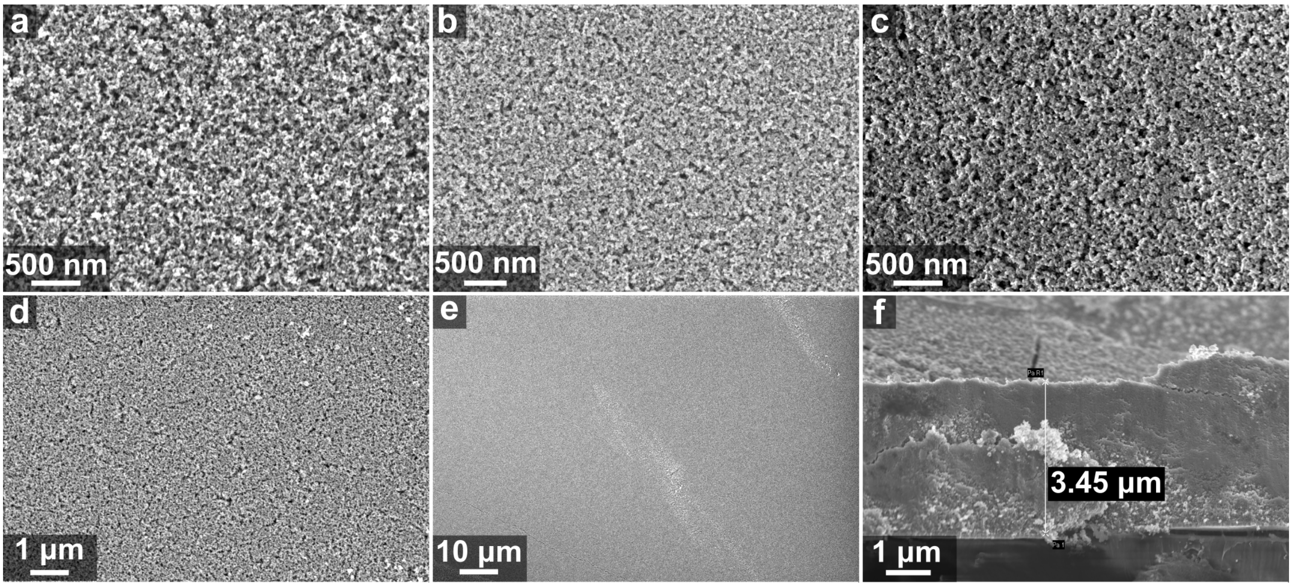

Figure S3: Scanning electron microscopy (SEM) images of mesoporous TiO_2: hotplate-annealed on FTO glass at 25k× (a), FIRA-annealed on FTO glass at 21k× (b), FIRA-annealed on ITO–polyimide at 25k× (c), 10k× (d) and 1k× (e). A cross-sectional view of an m-TiO_2 film atop ITO/PI photoanode at 11.8k×.

3.2 Four-Terminal Sensing

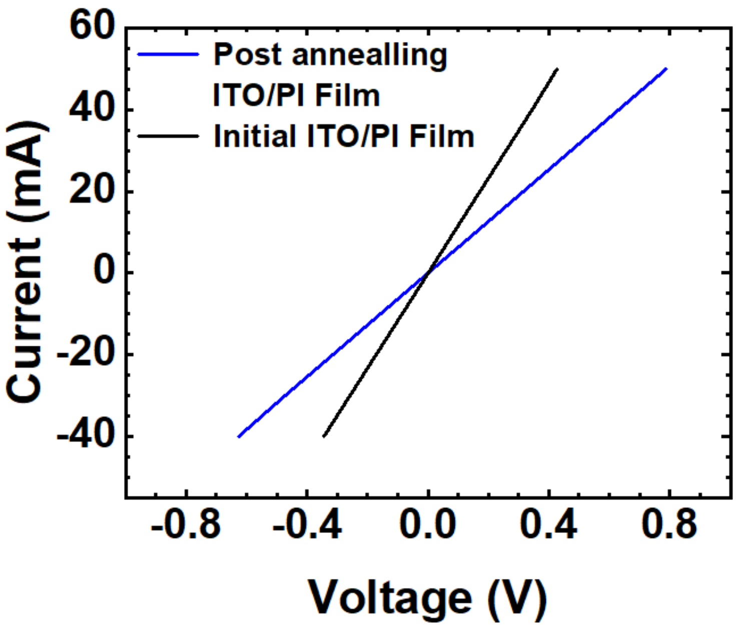

Figure S4: Four point probe sheet resistance measurement of ITO/Polyimide substrate pre and post FIRA annealing.

3.3 Thermal Modeling

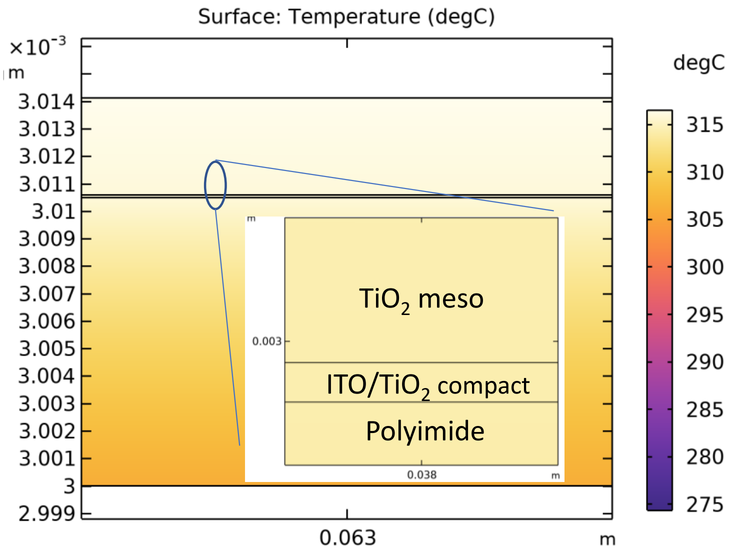

Figure S5: Thermal modeling of the active layers in a solar-cell photoanode stack under controlled irradiation.

The thermal modeling was performed with COMSOL Multiphysics (using the Heat Transfer in Solids interface), assuming bulk, temperature-independent properties and ideal planar interfaces for the ITO/TiO_2 compact, mesoporous TiO_2, and polyimide layers. While this stationary model cannot capture transient heating or nanoscale inhomogeneities, it provides otherwise inaccessible, depth-resolved temperature gradients across the stack, validated by FTIR binder loss and ITO resistance changes, and is essential for optimizing FIRA protocols where direct measurement is unfeasible.

3.4 UV-Vis Spectroscopy

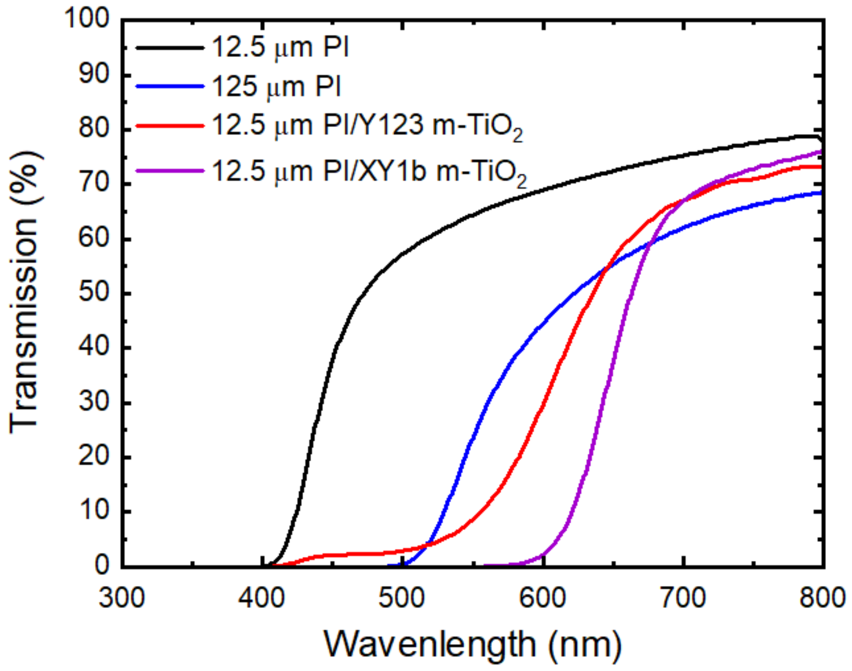

Figure S6: UV-Vis spectroscopy in transmission mode of PI films with thicknesses of 12.5 and 125 μm, and comparison of m-TiO_2 layers on top of a 12.5 μm PI film sensitized with XY1b or Y123 dyes.

4 Photovoltaic Characterization

4.1 Current Density-Voltage Data

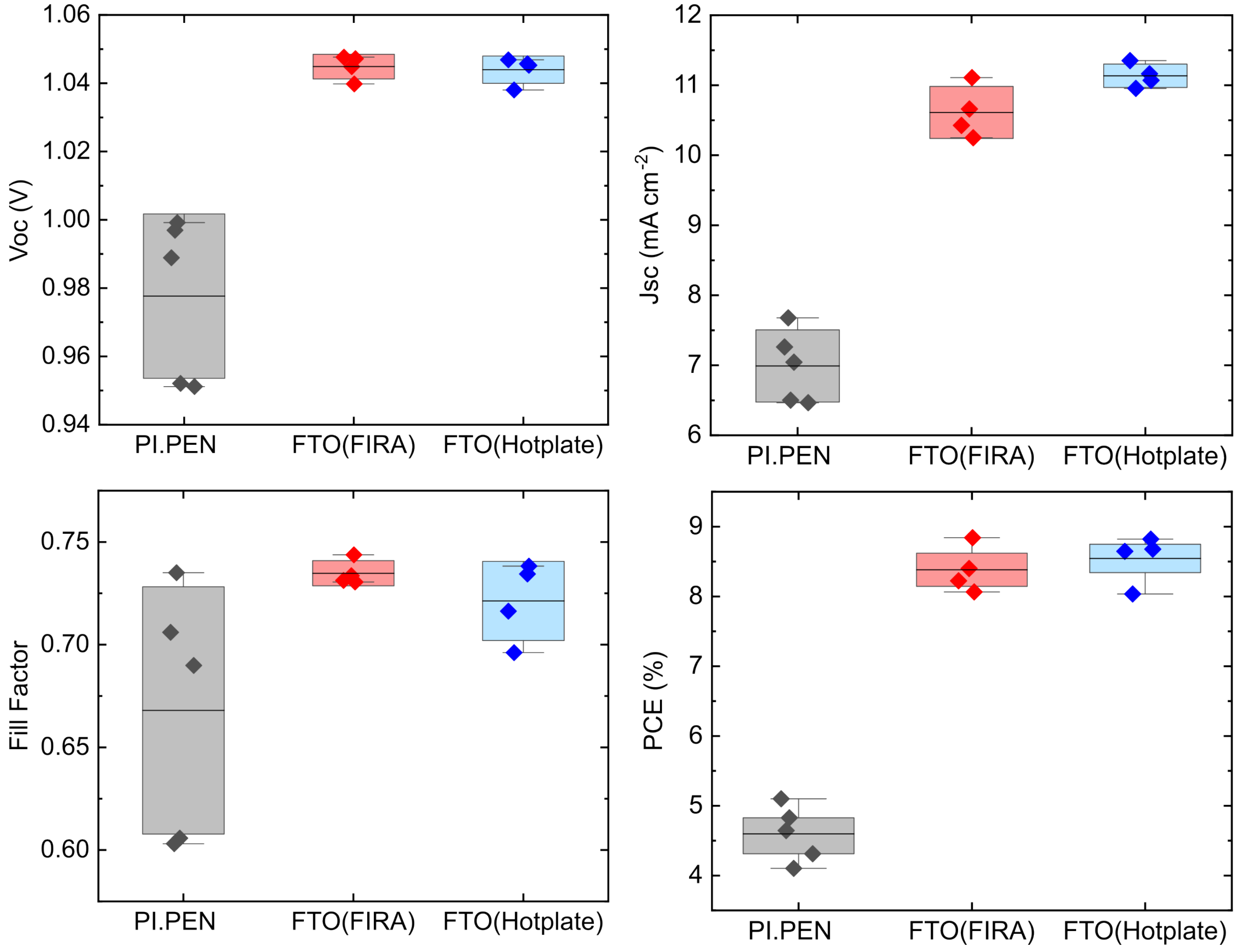

Figure S7: Overlap box plot of the distribution of the four J-V parameters (V_{OC}, J_{SC}, FF and PCE) at AM1.5G illumination. Whiskers show outliers, the standard deviation is represented by the colored box, and the average is represented by the central horizontal line.

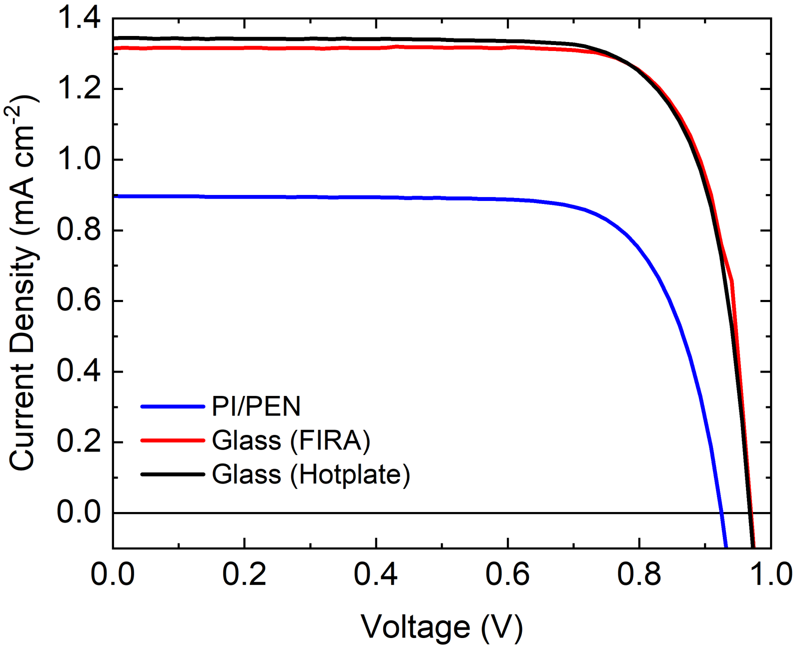

Figure S8: Current density-voltage characterization at 12% sun illumination.

Table S2: Current density-voltage characterization data for champion devices and average of 5 (PI/PEN) or 4 (glass) devices at full AM1.5G illumination, and for champion devices at 12% AM1.5G illumination.

Device	Condition	V_{OC} (V)	J_{SC} (mA cm^{-2})	FF	PCE (%)
PI/PEN	Champion	0.989	7.26	0.706	5.10
	Average	0.98 ± 0.2	7.0 ± 0.5	0.67 ± 0.06	4.6 ± 0.4
	12% sun	0.923	0.90	0.752	5.38
Glass “FIRA”	Champion	1.040	11.11	0.744	8.84
	Average	1.045 ± 0.004	10.6 ± 0.4	0.735 ± 0.006	8.4 ± 0.3
	12% sun	0.968	1.33	0.787	9.00
Glass “Hotplate”	Champion	1.046	11.16	0.734	8.82
	Average	1044 ± 0.004	11.1 ± 0.2	0.72 ± 0.02	8.5 ± 0.1
	12% sun	0.966	1.36	0.770	8.88

4.2 IPCE and UV-Vis Transmission

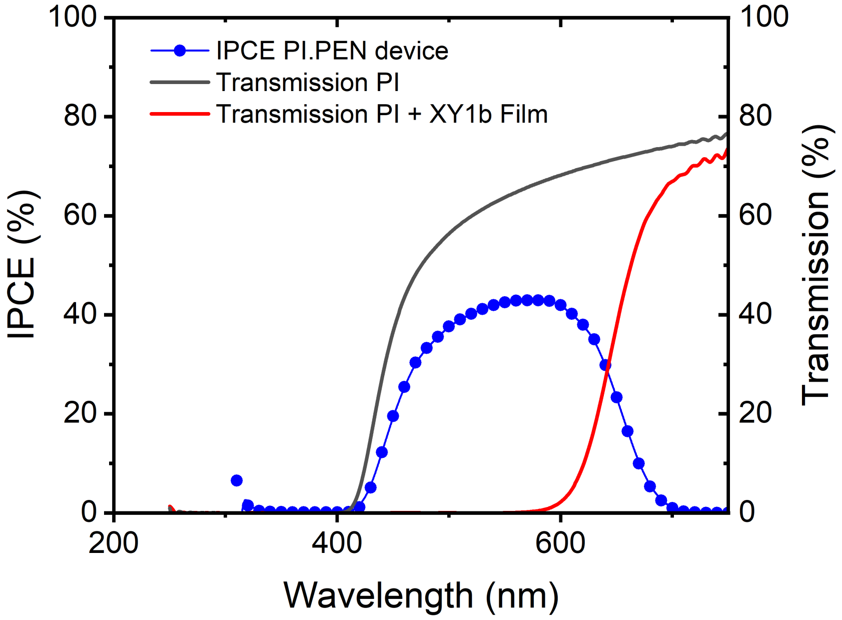

Figure S9: Combined plot of the IPCE spectrum of a PI/PEN device and of the UV-Vis spectrum in transmission mode of a PI film with and without an XY1b-sensitized m-TiO_2 layer.

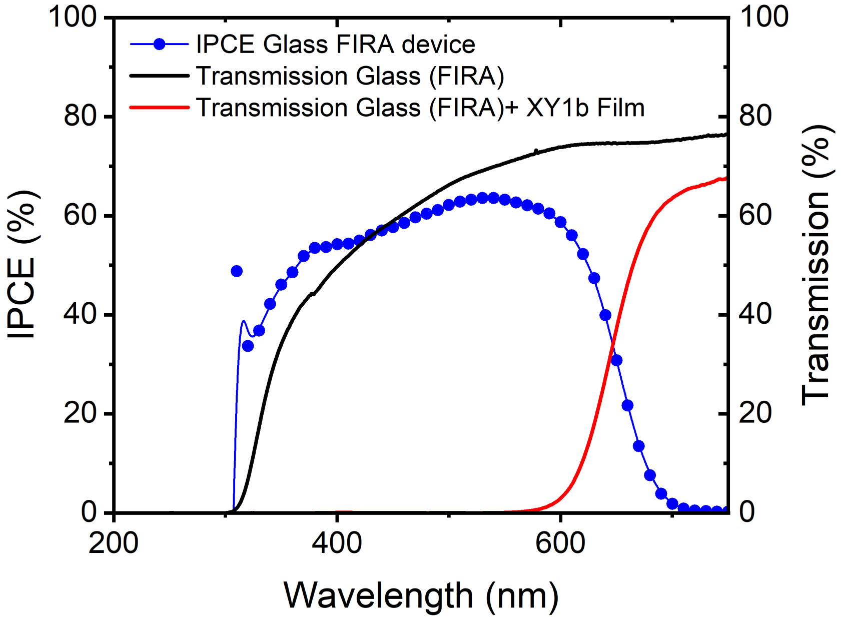

Figure S10: Combined plot of the IPCE spectrum of a glass "FIRA" device and of the UV-Vis spectrum in transmission mode of a glass substrate with and without an XY1b-sensitized m-TiO_2 layer.

4.3 Electrochemical Impedance Spectroscopy

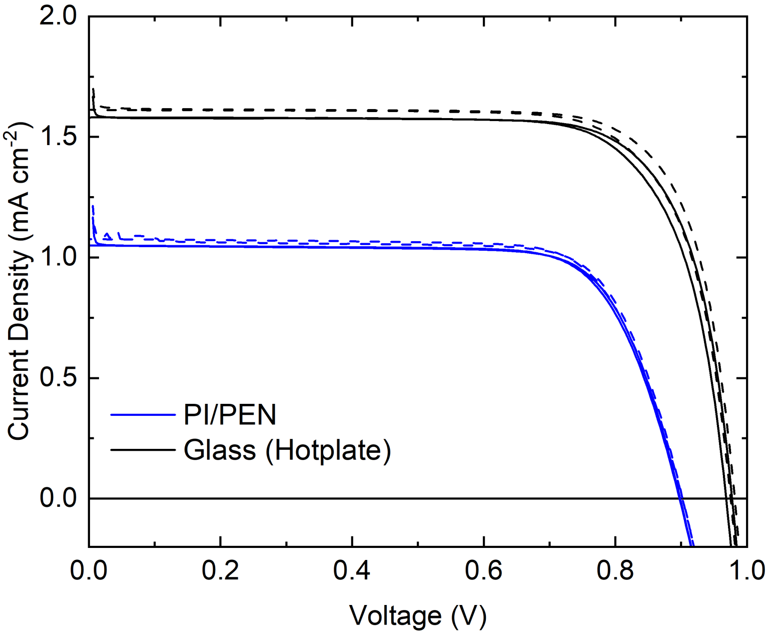

Figure S11: J-V curves measured via cyclic voltammetry using the Biologic potentiostat before (dash line) and after (full line) electrochemical impedance measurements under LED illumination.

Table S3: Extracted parameters from electrochemical impedance spectra on the DSC devices of Figure 3f, measured at the V_{OC} of Figure S11.

Parameter	PI/PEN	Glass “Hotplate”
R_s (Ω cm^2)	29.69	1.70
R_{trans} (Ω cm^2)	19.01	0.43
R_{TiO_2} (Ω cm^2)	41.08	47.50
C_{TiO_2} (10^{-4} F cm^{-2})	5.62	12.42
η	0.93	0.91
R_{ct} (Ω cm^2)	4.59	0.07
$Z_{CPE, CE}$ β	0.81	0.99
$Z_{CPE, CE}$ Q (10^{-4} Ω^{-1} cm^{-2} s$^{\beta}$)	0.33	4.63
Z_W R_W (Ω cm^2)	6.61	8.87
Z_W T_W (10^{-2} s)	7.15	3.51

4.4 Square-Wave Modulations Measurements under Illumination

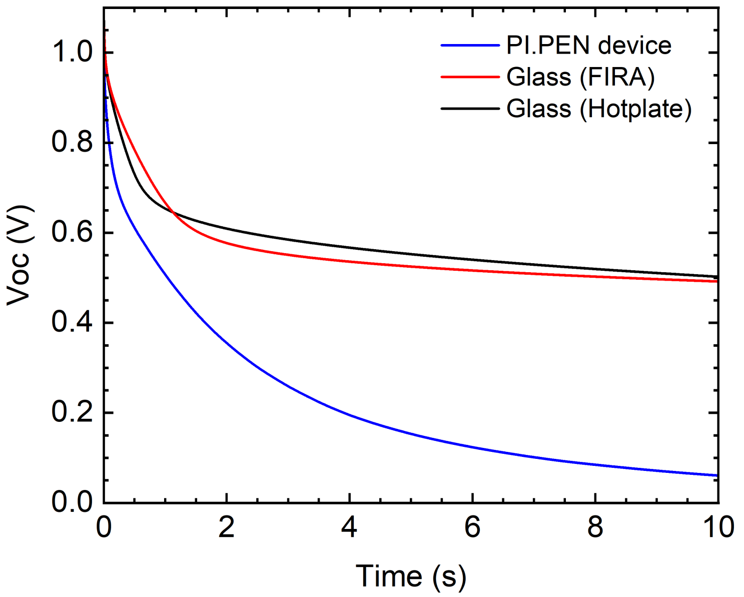

Figure S12: V_{OC} against time after LED illumination switch off.

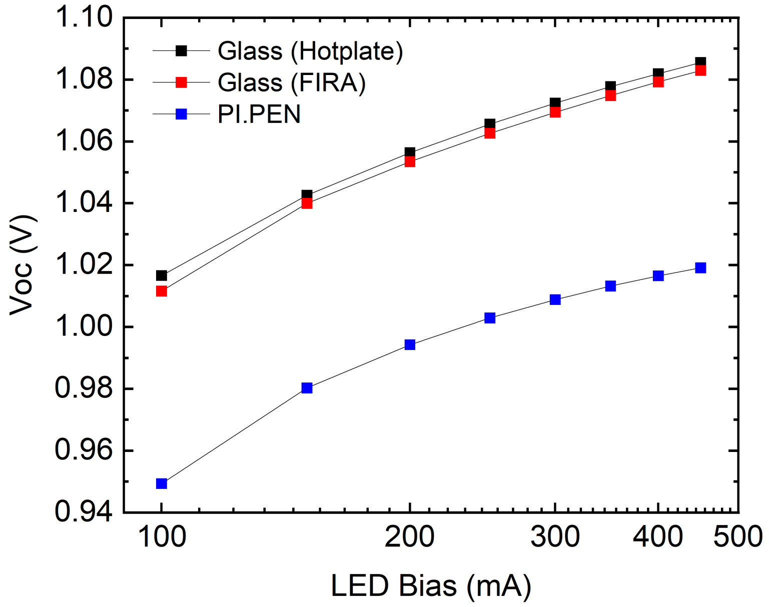

Figure S13: V_{OC} against applied current to LED light to control illumination intensity.

5 Life Cycle Assessment

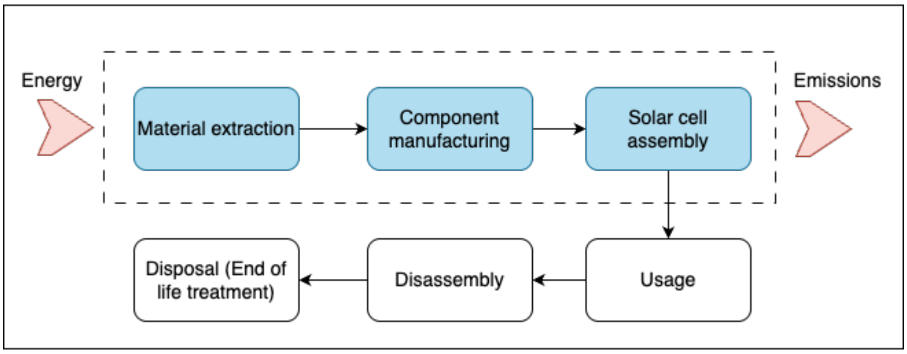

Figure S14: System boundary (dotted line) considered for the cradle-to-gate life cycle analysis.

Table S4: Absolute values (per cm^2) for the different impact categories based on the differing steps in the complete fabrication of the rigid DSCs for Figure 4a

Categories	FIRA	Hotplate
Ecosystem quality (species.yr)	2.91E-12	1.30E-12
Human health (DALYs)	1.87E-09	8.40E-10
Natural resources (USD 2013)	4.36E-05	1.96E-05
Global warming potential (kg CO2-Eq)	5.81E-04	2.60E-04
Cumulative energy demand (MJ-Eq)	1.23E-01	5.51E-02

Table S5: Absolute values (per cm^2) for the different impact categories based on the differing steps in the complete fabrication of FIRA-treated DSCs for Figure 4b.

Categories	Rigid	Flexible
Ecosystem quality (species.yr)	6.36E-08	4.88E-12
Human health (DALYs)	2.78E-06	3.14E-09
Natural resources (USD 2013)	5.34E-05	7.90E-05
Global warming potential (kg CO2-Eq)	6.23E-04	9.77E-04
Cumulative energy demand (MJ-Eq)	3.08E-02	2.02E-01

Table S6: Life cycle inventories (per cm^2) for differing steps in the fabrication of FIRA-treated and Hotplate-treated rigid substrates.

Sample	Processing steps	Input/Outputs	Amount	Unit
FIRA-treated	c-TiO_2 and mp-TiO_2 annealing	Electricity	1.96E-02	kWh
	c-TiO_2 and m-TiO_2 annealing	Cooling water pumping	4.08E-05	kWh
Hotplate-treated	c-TiO_2 and m-TiO_2 annealing	Electricity	8.79E-03	kWh

Table S7: Life cycle inventories (per cm^2) for differing steps in the fabrication of FIRA-treated rigid and flexible substrates

Sample	Processing steps	Input/Outputs	Amount	Unit
Flexible	Front substrate	12.5 μm PI[a]	1.59E-06	kg
	Front electrode	100 nm ITO	7.14E-08	kg
	Back electrode	300 nm ITO	2.14E-07	kg
	Back substrate	125 μm PEN	1.66E-05	kg
Rigid	Front substrate and electrode	400 nm FTO coated glass (4 mm)	2.50E-03	kg
	Back substrate and electrode	400 nm FTO coated glass (3 mm)	1.88E-03	kg

[a] Polyetherimide (a special class of PI) is assumed for the calculation.

6 Supplemental Experimental Procedures

6.1 FIRA annealing

The FIRA process utilizes a broadband 0.7–1.2 μm lamp array (24 kW m^{-2} peak irradiance) positioned about 8 cm above the sample being annealed. A water-cooled copper heat sink, maintained at 18 °C, is placed at the bottom of the chamber.

6.2 UV-Vis Spectroscopy

UV-Vis spectra were measured using a 1050+ Lambda spectrophotometer (PerkinElmer, with baseline correction). The transmission mode measurements were conducted using an integrating sphere with the substrates mounted to the sample holder.

6.3 4-point probe

Sheet resistance of the ITO-coated polyimide was measured with a home-built 4-point probe comprising a spring-loaded collinear probe head (tungsten needles, ≈1.0 mm tip spacing, ≈100 μm tip radius), a Keithley source/measure unit (Keithley 2450 SMU, current source, voltage sense), and an in-house control/DAQ software (Python + PyVISA). The probe head is mounted on a manual micrometer-driven Z stage. Measurements used a DC current sweep (10 μA–10 mA, auto-range), 4-wire sensing, current-reversal averaging to suppress thermoelectric offsets, and contact-check logic (dV/dI linearity, contact resistance threshold). Sheet resistance of the FTO glass was measured with an Ossila 4-point probe instrument (Ossila, Sheffield, UK) equipped with gold-plated probes with spacing of 1.27 mm and radius of 0.24 mm.

6.4 Current density–voltage and IPCE characterization

The photoactive area of 0.28 cm^2 of the dye sensitized solar cells was masked with an aperture area of 0.158 cm^2 for both J-V and IPCE measurements. Devices were measured under a 450 W Xenon light source of the Oriel solar simulator, equipped with a Schott K113 Tempax sunlight filter (Praezisions Glas & Optik GmbH) to match the emission spectrum of the lamp to the AM1.5G standard. The light intensity was determined using a calibrated Si reference diode equipped with an infrared cutoff filter (KG-3, Schott). The attenuated light intensity of 12% Sun was obtained by using a metal mesh. Results were recorded by a Keithley 2400 source meter. IPCE spectra were recorded with a commercial

apparatus (Arkeo-Ariadne, Cicci Research s.r.l.) based on a 300 W Xenon lamp. The shunt resistance was calculated from the inverse slope of the J-V curve near short circuit, while the series resistance from the inverse slope of the JV curve near open circuit, both under AM1.5G illumination.

6.5 Transient Current and Voltage Measurements ("Toolbox")

Electron lifetime measurements were performed using a white LED (Luxeon Star 1 W) as light source. Current traces were recorded with a 16-bit resolution digital acquisition board (National Instruments) and transport times were determined by monitoring photocurrent transients at different light intensities upon applying a small square wave modulation to the base light intensity. The photocurrent responses were fitted using first-order kinetics to obtain time constants. For the electron lifetime plot in Figure 3d, the electrochemical potential in the TiO_2 under open circuit (OC) conditions is calculated as follows: $E_{F,TiO2} = E_{redox} - V_{OC}$.[S8] E_{redox} of $Cu(tmby)_2$ electrolyte is reported.[S9]

6.6 Electrochemical Impedance Spectroscopy

Electrochemical impedance spectra for the full DSC devices were recorded using a Biologic VSP potentiostat under illumination with a white LED (Luxeon Star 1 W) and a voltage applied to match the V_{OC} of the device. The set voltage was applied for 100 s to stabilize the current and voltage before measurement start, which was conducted in the frequency range between 1 MHz and 100 mHz with a sinus amplitude of 20 mV s^{-1} (Vrms 14.14). The resulting output was fitted to an equivalent circuit model utilizing the Bisquert transmission line model with Zview (Scribner).

6.7 Scanning Electron Microscopy

SEM analyses were performed using a Zeiss GeminiSEM 300 at an acceleration voltage of 10 kV in Variable Pressure (VP) mode. Imaging was conducted using an Everhart-Thornley secondary electron (SE) detector. Additional configurations included the Inlens (SE) detector for enhanced imaging analysis.

6.8 Thermal Modeling

The thermal modeling was performed with COMSOL Multiphysics (using the Heat Transfer in Solids interface). A 2D geometry was constructed to represent the layered electrode structure and the layers between the sintered borosilicate glass, PI/ITO/TiO_2 compact, and a TiO_2 mesoporous layer. Each layer was assigned experimentally measured or literature-sourced thermal properties (thermal conductivity, density, and heat capacity). A stationary heat-transfer analysis was conducted by applying a prescribed heat flux at the top surface and including convective/radiative cooling at the outer boundaries. The resulting temperature field was solved iteratively. The script of the used method is available upon request.

6.9 Fourier Transform Infrared (FTIR) Spectroscopy

FITR measurements of the m-TiO_2 pre and post NIR annealing were conducted with a Spectrum Two FT-IR Spectrometer (Perkin Elmer, UK) equipped with a $LiTaO_3$ detector in universal attenuated total reflectance configuration.

6.10 Life Cycle Assessment

The analysis was based on life cycle inventory data collected from the experiments while including the effect of transportation of raw materials to Switzerland. For impacts of transportation, an average distance of 700 km (100 km by lorry and 600 km by train) was assumed for sourcing the raw materials from anywhere in Europe. Moreover, no waste treatment was considered, thus assuming 100% process efficiency. For the materials that are not present in the Ecoinvent database, stoichiometric reactions were assumed for their synthesis based on the existing literature. The impact characterization for raw materials and energy was obtained from the Ecoinvent database version 3.9.1 (2022) using the in-built "Allocation, cut-off by classification" strategy.

Supplemental References

S1 Wu, C.-T.; Kuo, H.-P.; Tsai, H.-A.; Pan, W.-C. Rapid Dye-Sensitized Solar Cell Working Electrode Preparation Using Far Infrared Rapid Thermal Annealing. *Applied Energy* **2012**, *100*, 138–143.

S2 Carnie, M. J.; Charbonneau, C.; Barnes, P. R. F.; Davies, M. L.; Mabbett, I.; Watson, T. M.; O'Regan, B. C.; Worsley, D. A. Ultra-Fast Sintered TiO2 Films in Dye-Sensitized Solar Cells: Phase Variation, Electron Transport and Recombination. *Journal of Materials Chemistry A* **2013**, *1*, 2225–2230.

S3 Watson, T.; Mabbett, I.; Wang, H.; Peter, L.; Worsley, D. Ultrafast near Infrared Sintering of TiO2 Layers on Metal Substrates for Dye-Sensitized Solar Cells. *Prog. Photovolt. Res. Appl.* **2011**, *19*, 482–486.

S4 Hooper, K.; Carnie, M.; Charbonneau, C.; Watson, T. Near Infrared Radiation as a Rapid Heating Technique for TiO2 Films on Glass Mounted Dye-Sensitized Solar Cells. *International Journal of Photoenergy* **2014**, *2014*, 953623.

S5 Yang, H.; Liu, W.; Xu, C.; Fan, D.; Cao, Y.; Xue, W. Laser Sintering of TiO2 Films for Flexible Dye-Sensitized Solar Cells. *Applied Sciences* **2019**, *9*, 823.

S6 Sánchez, S.; Jerónimo-Rendon, J.; Saliba, M.; Hagfeldt, A. Highly Efficient and Rapid Manufactured Perovskite Solar Cells via Flash InfraRed Annealing. *Materials Today* **2020**, *35*, 9–15.

S7 Cao, Y.; Liu, Y.; Zakeeruddin, S. M.; Hagfeldt, A.; Grätzel, M. Direct Contact of Selective Charge Extraction Layers Enables High-Efficiency Molecular Photovoltaics. *Joule* **2018**, *2*, 1108–1117.

S8 Feldt, S. M.; Lohse, P. W.; Kessler, F.; Nazeeruddin, M. K.; Grätzel, M.; Boschloo, G.; Hagfeldt, A. Regeneration and recombination kinetics in cobalt polypyridine based dye-sensitized solar cells, explained using Marcus theory. *Physical Chemistry Chemical Physics* **2013**, *15*, 7087–7097.

S9 Saygili, Y.; Söderberg, M.; Pellet, N.; Giordano, F.; Cao, Y.; Muñoz-García, A. B.; Zakeeruddin, S. M.; Vlachopoulos, N.; Pavone, M.; Boschloo, G.; Kavan, L.; Moser, J.-E.; Grätzel, M.; Hagfeldt, A.; Freitag, M. Copper Bipyridyl Redox Mediators for Dye-Sensitized Solar Cells with High Photovoltage. *Journal of the American Chemical Society* **2016**, *138*, 15087–15096.
